# Sex-Specific Risks of Occupational Distress in Physicians Compared With the General Population

**DOI:** 10.1001/jamanetworkopen.2025.40060

**Published:** 2025-10-29

**Authors:** Tait D. Shanafelt, Liselotte N. Dyrbye, Christine Sinsky, Michael Tutty, Hanhan Wang, Lindsey E. Carlasare, Colin P. West, Mickey Trockel

**Affiliations:** 1 Deparment of Medicine, Stanford University School of Medicine, Palo Alto, California; 2 Department of Medicine, University of Colorado School of Medicine, Denver; 3 American Medical Association, Chicago, Illinois; 4 WellMD & WellPhD Center, Stanford University, Palo Alto, California; 5 Department of Medicine, Mayo Clinic, Rochester, Minnesota; 6 Department of Psychiatry and Behavioral Health, Stanford University, Palo Alto, California

## Abstract

This survey study examines whether disparities in risk of burnout and low satisfaction with work-life integration by sex are similar among physicians compared with US workers in other fields.

## Introduction

Physicians have been found to have increased risk of burnout and lower satisfaction with work-life integration (WLI) vs workers in other fields. The heightened risk of distress seems confined to occupational dimensions, with no documented increased risk of depression.^1^ Research also indicates that women, whether they are physicians or working in other fields, face a higher risk of occupational burnout vs men.^1,2,3^

## Methods

To explore whether disparity in risk of burnout and low satisfaction with WLI by sex was similar in physicians vs US workers in other fields, we analyzed data from a national survey study of physicians and US workers over the last 12 years. Institutional review board review and approval was provided by the Mayo Clinic (2011, 2014, 2017, 2020, and 2023), Stanford University (2017, 2020, and 2023), and the University of Illinois Chicago (2023) across survey years. Return of a completed survey was considered implied consent. This study follows the AAPOR reporting guideline for survey studies. Details regarding survey methods are provided in the eAppendix in Supplement 1, and occupations of the comparison population are shown in the eTable in Supplement 1.

## Results

Overall, 25 248 physicians (median [IQR] age, 52 [43-59] years; 9794 [39.0%] female) and 20 283 US workers (median [IQR] age, 50 [41-57] years; 9560 [47.1%] female) participated in the 2011, 2014, 2017, 2020, and 2023 surveys. In multivariable analysis adjusting for age, relationship status, hours worked per week, practice setting, and specialty, female physicians were at higher risk for burnout than their male colleagues at 4 of 5 survey time points, with odds ratios (ORs) ranging from 1.26 (95% CI, 1.11-1.42) to 1.34 (95% CI, 1.17-1.54) between 2014 and 2023 (Table 1). Among US workers, after adjusting for age, relationship status, and hours worked per week, female individuals were at higher risk for burnout than male individuals at 3 of 5 survey time points, with ORs ranging from 1.24 (95% CI, 1.09-1.41) to 1.45 (95% CI, 1.20-1.76) between 2017 and 2023.

**Table 1.** Multivariable Analysis of Factors Associated With Burnout and Satisfaction With WLI at Each Time Point

Variable	OR (95% CI), female vs male individuals				
	2011	2014	2017	2020	2023
Burnout^a^					
Physicians^b^	1.06 (0.94-1.20)	1.26 (1.11-1.42)^c^	1.34 (1.17-1.54)^c^	1.27 (1.12-1.44)^c^	1.24 (1.10-1.40)^c^
US workers^d^	1.11 (0.97-1.27)	1.10 (0.97-1.25)	1.24 (1.09-1.41)^c^	1.45 (1.20-1.76)^c^	1.37 (1.14-1.63)^c^
Satisfaction WLI					
Physicians^b^	0.65 (0.57-0.74)^c^	0.69 (0.61-0.79)^c^	0.54 (0.46-0.62)^c^	0.63 (0.55-0.71)^c^	0.60 (0.53-0.68)^c^
US workers^d^	0.99 (0.88-1.11)	0.77 (0.68-0.87)^c^	0.92 (0.81-1.04)	0.82 (0.68-0.97)^c^	0.73 (0.62-0.86)^c^

Abbreviations: OR, odds ratio; WLI, work-life integration.

^a^ Burnout was assessed using the single-item measures for emotional exhaustion and depersonalization adapted from the full Maslach Burnout Inventory. For more information, see the eAppendix in Supplement 1.

^b^ Adjusted for age, relationship status, hours worked per week, specialty, and practice setting.

^c^ *P* < .05.

^d^ Adjusted for age, relationship status, hours worked per week, and education levels.

Female physicians had lower odds of satisfaction with WLI vs male physicians at all 5 survey time points (OR range, 0.54 [95% CI, 0.46-0.62] to 0.69 [95% CI, 0.61-0.79]). Female US workers had lower odds of satisfaction with WLI vs male workers at 3 of 5 survey time points (2014 OR, 0.77 [95% CI, 0.68-0.87]; 2020 OR, 0.82 [95% CI, 0.68-0.97]; 2023 OR, 0.73 [95% CI, 0.62-0.86]).

We subsequently analyzed pooled data across all survey time points to evaluate the associations of sex, being a physician, and the interaction between sex and being a physician with burnout and WLI (Table 2). Female individuals had 15% higher odds of burnout (OR, 1.15; 95% CI, 1.11-1.20) after adjusting for age, relationship status, hours worked per week, and survey year. Physicians had 63% higher odds of burnout (OR, 1.63; 95% CI, 1.56-1.71) after adjusting for these factors. The sex disparity in burnout risk was not statistically significantly different among physicians than other US workers.

**Table 2.** Multivariable Analysis of Physicians and Workers in Other Fields, 2011 to 2023, Adjusting for Age, Relationship Status, and Hours Worked Per Week

Variable	Burnout		Satisfaction WLI	
	OR (95% CI)	*P* value	OR (95% CI)	*P* value
Female (vs male)	1.15 (1.11-1.20)	<.001	0.84 (0.79-0.89)	<.001
Physicians (vs other US workers)	1.63 (1.56-1.71)	<.001	0.80 (0.76-0.85)	<.001
Hours worked per week	1.02 (1.02-1.02)	<.001	0.95 (0.95-0.96)	<.001
Age, y (vs <35 y)				
35-44	0.95 (0.87-1.03)	.20	0.89 (0.82-0.97)	.01
45-54	0.96 (0.88-1.04)	.26	0.90 (0.83-0.98)	.02
55-64	0.76 (0.70-0.82)	<.001	0.99 (0.92-1.08)	.88
Relationship status (vs single)				
Married	0.72 (0.69-0.76)	<.001	1.33 (1.26-1.40)	<.001
Partnered	0.90 (0.82-0.99)	.04	1.16 (1.05-1.28)	.004
Widowed	0.86 (0.71-1.04)	.12	1.20 (0.99-1.45)	.05
Survey year (vs 2011)				
2014	1.29 (1.22-1.37)	<.001	1.01 (0.95-1.07)	.74
2017	1.05 (0.99-1.12)	.13	1.09 (1.03-1.16)	.005
2020	0.84 (0.79-0.90)	<.001	1.16 (1.09-1.23)	<.001
2023	0.98 (0.92-1.04)	.50	1.02 (0.96-1.10)	.46
Interaction between being both female and a physician	NA^a^	NA^a^	0.80 (0.74-0.87)^b^^,^^c^	<.001

Abbreviations: NA, not applicable; OR, odds ratio; WLI, work-life integration.

^a^ The interaction term was removed from the model after it was tested and was found to be not significant (OR, 0.96; 95% CI, 0.88-1.04).

^b^ Combined effect of being female (OR, 0.84; 95% CI, 0.79-0.89) and interaction effect of sex and being a physician (OR, 0.80; 95% CI, 0.74-0.87) translates into female individuals having 33% lower odds (0.84 × 0.80 = 0.67) of being satisfied with WLI, among physicians.

^c^ Combined effect of being a physician (OR, 0.80; 95% CI, 0.76-0.85) and interaction effect of sex and being a physician (OR, 0.80; 95% CI, 0.74-0.87) translates into female physicians having 36% lower odds (0.80 × 0.80 = 0.64) of being satisfied with WLI, compared with female individuals who are workers in other fields.

For WLI, a statistically significant interaction was observed between being both female and a physician (OR, 0.80; 95% CI, 0.74-0.87), indicating the sex disparity in odds of satisfaction with WLI was greater among physicians than among other US workers. Female physicians had 33% lower odds of being satisfied with WLI than male physicians after adjusting for other factors compared with the 16% (OR, 0.84; 95% CI, 0.79-0.89) lower odds of satisfaction with WLI among female workers outside of medicine vs male workers outside medicine (Table 2). Female physicians had 36% lower odds of satisfaction with WLI vs other female US workers, while male physicians had 20% lower odds of satisfaction with WLI vs other male US workers.

## Discussion

This survey study found that the sex disparity in occupational burnout among physicians is similar to that observed across the US workforce. In contrast, the sex disparity in satisfaction with WLI among physicians is larger than that observed across the US workforce even after adjusting for work hours. This analysis is limited by survey response rates and potential response bias. Potential contributing factors to greater challenges with WLI among female physicians may include professional norms, uneven distribution of home and family responsibilities, mistreatment experiences, and different patient and staff expectations for women in medicine.^4^ Recent studies have also indicated that career support from physicians’ spouse or partner influences satisfaction with WLI^5^ and that an organizationally sponsored couples’ health promotion program can mitigate the adverse impact of work on physicians’ personal relationships.^6^ Additional studies are needed to determine why sex-specific disparities in WLI are larger for physicians than the general workforce and to address the factors that contribute to this disparity.

## Notes

Supplement 1. eAppendix. Supplemental Methods eTable. Occupation of US Workers in Comparison Population eReferences

Supplement 2. Data Sharing Statement
