# Supplementary material for: Sex-Specific Risks of Occupational Distress in Physicians Compared With the General Population
Source: JAMA Netw Open. 2025 Oct 29;8(10):e2540060. doi: 10.1001/jamanetworkopen.2025.40060 (PMC12573027; doi:10.1001/jamanetworkopen.2025.40060)
Supplement: Supplement 1. — eAppendix. Supplemental Methods eTable. Occupation of US Workers in Comparison Population eReferences [file jamanetwopen-e2540060-s001.pdf]

Supplemental Online Content

Shanafelt TD, Dyrbye LN, Sinsky C, et al. Sex-specific risks of occupational distress in physicians compared with the general population. *JAMA Netw Open*. 2025;8(10):e2540060.
doi:10.1001/jamanetworkopen.2025.40060

eAppendix. Supplemental Methods
eTable. Occupation of US Workers in Comparison Population
eReferences

This supplemental material has been provided by the authors to give readers additional information about their work.

© 2025 Shanafelt TD et al. *JAMA Network Open*.

eAppendix. Supplemental Methods

As previously described, cross-sectional surveys were administered in 2011, 2014, 2017, 2020 and 2023.[1-5] The methodology was similar at all time points with the exception that the 2011 and 2014 surveys were exclusively online while a combination of online and mailed surveys was used at the other time points. Financial incentives were offered to a subset of survey participants in 2020 and 2023.

<u>Participants:</u>

Samples of 83,291-95,079 physicians from across specialties were assembled at each timepoint using the American Medical Association Physician Professional Data file, a nearly complete record of all US physicians independent of AMA membership. Physicians in non-primary care specialties were oversampled at all timepoints to provide an adequate number of participants from less common specialties. Information on the dates of survey administration as well as the nuances and distinct aspects of administration in each survey year have been previously reported.[1-5]

Survey response rates across survey years ranged from 7.1%-26.7%. This variation in response rate by year was in part influenced by whether or not the online survey tracking software allowed determination of whether or not participants received the survey email. In accord with American Association for Public Opinion Research guidelines, in years where that information was available, those who opened at least 1 invitation e-mail were considered to have received an invitation to participate in the study.[6] In years where that information was not available, the total number of

© 2025 Shanafelt TD et al. *JAMA Network Open*.

delivered emails was used as the number of individuals invited to participate in the study (i.e.,a conservative approach).

Secondary surveys with financial incentives were conducted in samples of non-responders to the primary survey in 2017, 2020 and 2023 to evaluate response bias. No statistically significant differences in burnout or satisfaction with work-life integration were observed between participants in the secondary surveys of non-responders and primary survey participants suggesting burnout and satisfaction with WLI among participants in the primary surveys were representative of US physicians.[3-5]

At each time point, a probability-based sample of employed US adults was surveyed using the KnowledgePanel®.[7] Employed responders across all levels of education who were age 29-65 at the time of the survey were used as a comparator sample for non-retired physicians in the same age range at each survey timepoint. Detailed information regarding highest level of education completed and occupation at each survey timepoint have been previously reported.[1-5] Information on occupation for the US worker comparison group in the present manuscript is provided in the Supplemental Table. IRB review and approval was provided by the Mayo Clinic (2011, 2014, 2017, 2020, 2023), Stanford University (2017, 2020, 2023), and the University of Illinois Chicago (2023) across survey years. For physician surveys, participation was voluntary, all the responses were anonymous, and return of completed survey was considered implied consent.

<u>Survey Measures:</u>

© 2025 Shanafelt TD et al. *JAMA Network Open*.

Both physician participants and population controls provided basic demographic information and information regarding hours worked each week. Individuals in the general workforce provided information on their occupation and highest level of education completed. Physicians provided additional information regarding professional characteristics such as specialty and practice setting. In the 2011, 2014, 2017 and 2020 physician surveys, gender/sex was assessed by asking participants "What is your gender?" (response options 2011 and 2014: "male", "female"; response options 2017 and 2020: male, female, other). In the 2023 survey, gender was assessed by asking participants "Gender: how do you identify?" (response options: man, woman, non-binary, another gender identify [free text], prefer not to say). For the present analysis, those who responded "male" and "man" or "female" and "woman" were grouped as "male" and "female" and included in the analysis.

For the present analysis, burnout was measured using two single-item measures from the Maslach Burnout Inventory assessing emotional exhaustion (EE) and depersonalization (DP) used under license from Mind Garden, Inc. EE and DP as assessed by these two items have been found to have high correlation (areas under receiver operator characteristic [ROC] curve both $\geq$0.93) with the full-length EE and DP scales of the Maslach Burnout Inventory.[8,9] In accord with convention and prior reports, individuals indicated they experienced symptoms of EE and/or DP at least weekly or more often were considered to have at least one symptom of burnout.[8,9]

Satisfaction with work-life integration was assessed at all time points in both physicians and population samples by asking participants to rate their agreement with the statement, "My work schedule leaves me enough time for my personal/family life" (response options: strongly agree,

© 2025 Shanafelt TD et al. *JAMA Network Open*.

agree, neutral, disagree, or strongly disagree). Individuals who indicated they agreed or strongly agreed with this statement were considered to be satisfied with work-life integration.

Statistical methods:

Standard summary statistics for demographic characteristics, burnout and satisfaction with WLI have been reported in previous studies.[1-5] Multivariable logistic regressions were conducted to examine the association between sex and outcome variables (burnout and satisfaction with WLI). For physicians, this analysis adjusted for age, relationship status, hours worked per week, specialty and practice settings. For US workers in other fields, this analysis adjusted for age, relationship status, hours worked per week and highest level of education completed. The pooled multivariable logistic regressions combining physicians and US workers in other field conducted to examine the interaction between both sex and being a physician were adjusted for age, relationship status, hours worked per week and survey year. All tests were two-tailed, with a statistical significance threshold set at α=0.05. All analyses were performed using R Version 4.5.0 (R Foundation for Statistical Computing, 2025).

© 2025 Shanafelt TD et al. *JAMA Network Open*.

eTable: Occupation of US Workers in Comparison Population

	N (%)[a]
Professional[b]	1492 (52.1%)
Health Care[c]	104 (3.6%)
Service[d]	215 (7.5%)
Sales[e]	171 (6.0%)
Office and Administrative Support	264 (9.2%)
Farming, Forestry Fishing	14 (0.5%)
Precision Production, Craft and Repair[f]	183 (6.4%)
Transportation and Material	103 (3.6%)
Armed services	9 (0.3%)
Other	311 (10.9%)
Missing	1

[a] Age 29-65 actively employed at the time of the survey

[b] business/financial, management, computer/mathematical, architecture/engineering, lawyer/judge, life/physical/social sciences, community/social services, teacher non-university, teacher college/university, other
[c] nurse, pharmacist, paramedic, lab technician, nursing aide, orderly, dental assistant
[d] protective service, food preparation/service, building cleaning/maintenance, personal care/service
[e] sales representative, retails sales, other sales
[f] construction and extraction, installation/maintenance/repair, precision production (machinist, welder, backer, printer, tailor)

© 2025 Shanafelt TD et al. *JAMA Network Open*.

eReferences:

1. Shanafelt TD, Boone S, Tan L, et al. Burnout and satisfaction with work-life balance among US physicians relative to the general US population. Research Support, Non-U.S. Gov't. *Arch Intern Med*. Oct 8 2012;172(18):1377-85. doi:10.1001/archinternmed.2012.3199
2. Shanafelt TD, Hasan O, Dyrbye LN, et al. Changes in Burnout and Satisfaction With Work-Life Balance in Physicians and the General US Working Population Between 2011 and 2014. *Mayo Clin Proc*. Dec 2015;90(12):1600-13. doi:10.1016/j.mayocp.2015.08.023
3. Shanafelt TD, West CP, Sinsky C, et al. Changes in Burnout and Satisfaction With Work-Life Integration in Physicians and the General US Working Population Between 2011 and 2017. *Mayo Clin Proc*. Sep 2019;94(9):1681-1694. doi:10.1016/j.mayocp.2018.10.023
4. Shanafelt TD, West CP, Sinsky C, et al. Changes in Burnout and Satisfaction With Work-Life Integration in Physicians and the General US Working Population Between 2011 and 2020. *Mayo Clin Proc*. Mar 2022;97(3):491-506. doi:10.1016/j.mayocp.2021.11.021
5. Shanafelt TD, West CP, Sinsky C, et al. Changes in Burnout and Satisfaction With Work-Life Integration in Physicians and the General US Working Population Between 2011 and 2023. *Mayo Clin Proc*. Jul 2025;100(7):1142-1158. doi:10.1016/j.mayocp.2024.11.031
6. American association for public opinion research (AAPOR):. Standard Definitions: Final Dispositions of Case Codes and Outcome Rates for Surveys (revised 2016). 2016. Accessed 8/22/2016. http://www.aapor.org/AAPOR_Main/media/publications/Standard-Definitions20169theditionfinal.pdf
7. Ipsos. KnowledgePanel. Ipsos. Accessed 7/14/25, 2025. https://www.ipsos.com/en-us/solutions/public-affairs/knowledgepanel
8. West CP, Dyrbye LN, Satele DV, Sloan JA, Shanafelt TD. Concurrent validity of single-item measures of emotional exhaustion and depersonalization in burnout assessment. Research Support, Non-U.S. Gov't
Validation Studies. *Journal of general internal medicine*. Nov 2012;27(11):1445-52. doi:10.1007/s11606-012-2015-7
9. West CP, Dyrbye LN, Sloan JA, Shanafelt TD. Single item measures of emotional exhaustion and depersonalization are useful for assessing burnout in medical professionals. Comparative Study Research Support, Non-U.S. Gov't. *Journal of general internal medicine*. Dec 2009;24(12):1318-21. doi:10.1007/s11606-009-1129-z

© 2025 Shanafelt TD et al. *JAMA Network Open*.
