# Supplementary material for: Sex-Specific Risks of Occupational Distress in Physicians Compared With the General Population
Source: JAMA Netw Open. 2025 Oct 29;8(10):e2540060. doi: 10.1001/jamanetworkopen.2025.40060 (PMC12573027; doi:10.1001/jamanetworkopen.2025.40060)
Supplement: Supplement 2. — Data Sharing Statement [file jamanetwopen-e2540060-s002.pdf]

Data Sharing Statement

Shanafelt. Sex-Specific Risks of Occupational Distress in Physicians Compared With the General Population. *JAMA Netw Open*. Published October 29, 2025. doi:10.1001/jamanetworkopen.2025.40060

Data
Data available: No
